# Three Adult Cases of STAT1 Gain-of-Function with Chronic Mucocutaneous Candidiasis Treated with JAK Inhibitors

**DOI:** 10.1007/s10875-022-01351-0

**Published:** 2022-09-02

**Authors:** Emilie W. Borgström, Marie Edvinsson, Lucía P. Pérez, Anna C. Norlin, Sara L. Enoksson, Susanne Hansen, Anders Fasth, Vanda Friman, Olle Kämpe, Robert Månsson, Hernando Y. Estupiñán, Qing Wang, Tan Ziyang, Tadepally Lakshmikanth, Carl Inge E. Smith, Petter Brodin, Peter Bergman

**Affiliations:** 1 Department of Laboratory Medicine, Clinical Microbiology, Stockholm, Sweden; 2 Department of Infectious Diseases, Karolinska University Hospital, Stockholm, Sweden; 3 Department of Medical Sciences, Section of Infectious Diseases, Uppsala University Hospital, Uppsala, Sweden; 4 Department of Laboratory Medicine, Biomolecular and Cellular Medicine, Karolinska Institutet, Stockholm, Sweden; 5 Department of Clinical Immunology and Transfusion Medicine, Karolinska University Hospital, Stockholm, Sweden; 6 Department of Pediatrics, Institute of Clinical Sciences, Sahlgrenska Academy, University of Gothenburg, Gothenburg, Sweden; 7 Department of Infectious Diseases, Institute of Biomedicine, Sahlgrenska Academy, University of Gothenburg, Gothenburg, Sweden; 8 Experimental Endocrinology, Department of Medicine, Karolinska Institutet, Solna, Stockholm, Sweden; 9 Departamento de Ciencias Básicas, Universidad Industrial de Santander, 680002 Bucaramanga, Colombia; 10 Science for Life Laboratory, Department of Women’s and Children’s Health, Karolinska Institutet, Stockholm, Sweden; 11 Department of Laboratory Medicine, Translational Research Center Karolinska (TRACK), Stockholm, Sweden; 12 Department of Immunology and Inflammation, Imperial College London, London, UK

**Keywords:** JAK inhibitor, chronic mucocutaneous candidiasis, *STAT1*, mass cytometry, Olink

## Abstract

**Purpose:**

The aim of this study was to characterize clinical effects and biomarkers in three patients with chronic mucocutaneous candidiasis (CMC) caused by gain-of-function (GOF) mutations in the *STAT1* gene during treatment with Janus kinase (JAK) inhibitors.

**Methods:**

Mass cytometry (CyTOF) was used to characterize mononuclear leukocyte populations and Olink assay to quantify 265 plasma proteins. Flow-cytometric Assay for Specific Cell-mediated Immune-response in Activated whole blood (FASCIA) was used to quantify the reactivity against *Candida albicans*.

**Results:**

Overall, JAK inhibitors improved clinical symptoms of CMC, but caused side effects in two patients. Absolute numbers of neutrophils, T cells, B cells, and NK cells were sustained during baricitinib treatment. Detailed analysis of cellular subsets, using CyTOF, revealed increased expression of CD45, CD52, and CD99 in NK cells, reflecting a more functional phenotype. Conversely, monocytes and eosinophils downregulated CD16, consistent with reduced inflammation. Moreover, T and B cells showed increased expression of activation markers during treatment. In one patient with a remarkable clinical effect of baricitinib treatment, the immune response to *C. albicans* increased after 7 weeks of treatment. Alterations in plasma biomarkers involved downregulation of cellular markers CXCL10, annexin A1, granzyme B, granzyme H, and oncostatin M, whereas FGF21 was the only upregulated marker after 7 weeks. After 3 months, IFN-ɣ and CXCL10 were downregulated.

**Conclusions:**

The clinical effect of JAK inhibitor treatment of CMC is promising. Several biological variables were altered during baricitinib treatment demonstrating that lymphocytes, NK cells, monocytes, and eosinophils were affected. In parallel, cellular reactivity against *C. albicans* was enhanced.

**Supplementary Information:**

The online version contains supplementary material available at 10.1007/s10875-022-01351-0.

## Introduction

Chronic mucocutaneous candidiasis (CMC) is a clinical syndrome with inborn errors of IL-17 immunity, characterizing a group of primary immunodeficiencies with persistent inflammation in mucous membranes, caused by *Candida* species, most often *C. albicans*. The clinical picture of this syndrome is heterogeneous, with both chronic and acute infections, as well as autoimmune manifestations. In addition, cerebral aneurysms may occur, but are rare [1]. STAT1 gain of function (GOF) mutations are the most prevalent mutations in this syndrome [2, 3].

Signal transducers and activators of transcription (*STAT*s) are DNA-binding transcription factors of importance for intracellular signaling in immune cells. They are activated by Janus tyrosine kinases (JAK) and they carry a phosphotyrosine-binding SH2 domain [4]. *STAT1* is phosphorylated by cytoplasmic JAK1/2 following cytokine receptor stimulation by cytokine ligands (IFN-α/ß, IFN-ɣ, IFN-λ, and IL-27) [5, 6], which leads to *STAT1* homo- or heterodimerization and translocation of the complex into the cell nucleus with subsequent effects on target genes. The underlying mechanisms for STAT1 gain of activity in mononuclear cells in patients with CMC are not fully understood and may differ depending on the causative mutations. A large study on kindreds with CMC found that the mechanism involves a loss of nuclear dephosphorylation of STAT1 causing accumulation of the protein in the nucleus [2]. Increased STAT1 levels could thus compete with STAT3 for DNA-binding sites, which may cause decreased expression of STAT3-dependent genes in CMC [7]. Alternatively, STAT1 may directly induce a set of IFN-ɣ-induced genes, which could contribute to the pathology in patients with STAT1 GOF mutations [8]. STAT1 GOF and STAT3 loss-of-function (LOF) syndromes show common clinical signs including infectious and inflammatory manifestations, and a recent report describes the effect of ruxolitinib on *STAT3* dominant negative cells [9].

*STAT3* is mainly activated by the cytokines IL-6 [10], IL-21 [11], and IL-23 [12] following binding to their corresponding receptors. These cytokines are released by innate immune cells and by infected epithelial cells, e.g., during *Candida* infection [13]. *STAT3* regulates the development of adaptive immunity and differentiation of CD4^+^ T helper (Th) cells to Th17 cells [14]. Th17 cells subsequently produce IL-17A, IL-17F [15], and IL-22 which are important cytokines for mucosal immunity [16]. CMC is connected to severely impaired Th17 cell function [17] and loss of mucosal barriers against *Candida* species [18].

Since the CMC syndrome may have different etiologies, a detailed molecular diagnosis is essential to provide a personalized treatment strategy based on the individual’s genetic alteration. A few clinical studies, mainly in children, have presented patients with the STAT1 GOF syndrome treated with the JAK1/2 inhibitor ruxolitinib (*n* = 33 cases), and the clinical status was generally improved [19–31]. Ruxolitinib inhibits tyrosine kinase activity in target cells and diminishes subsequent phosphorylation and activation of STAT1 and is approved for use in polycythemia vera [32] and myelofibrosis [33, 34]. It is associated with a substantial risk of side effects when used in myelofibrosis, such as pneumonia, herpes zoster, and septicemia, as well as bone marrow suppression [35]. In a multi-center study by Forbes et al., adverse events in patients with either STAT1 or STAT3 GOF treated with JAK inhibitors ruxolitinib (*n* = 16) or tofacitinib (*n* = 1) were transient thrombocytopenia, elevated transaminases and bilirubin, and viral infections such as herpes zoster (*n* = 2), viral bronchitis (*n* = 4), and gastroenteritis (*n* = 1). Four patients died from uncontrolled inflammation and/or infection [25].

Baricitinib is another selective JAK1/2 inhibitor, which is approved for the treatment of rheumatoid arthritis (RA) [36] and atopic dermatitis (AD) [37]. The mechanism of action for baricitinib is similar to that of ruxolitinib, but baricitinib is a less potent JAK inhibitor, although slightly more selective for JAK1 and 2. Baricitinib increases the risk of upper respiratory tract infections and herpes simplex/zoster infections, but was not associated with a higher risk of serious infections in patients with RA and AD [38]. This was the rationale for testing baricitinib in the patients presented in this report [37, 39]. The first published case of STAT1 GOF treated with baricitinib had a beneficial outcome and no adverse events [40]. The second published case had no beneficial clinical effect of baricitinib, and treatment was stopped after 2 months [24].

Hematopoietic stem cell transplantation (HSCT) is a treatment option, which could be considered in monogenic immunodeficiency disorders. A study on 15 patients 1–33 years old with STAT1 GOF who underwent HSCT, showed donor engraftment in 74% and an overall survival of 40% [41]. The mortality could be lowered with reduced-intensity conditioning [42]. However, the benefit of HSCT may not outweigh the risks in most patients with STAT1 GOF syndrome.

Notably, the detailed immunological alterations caused by JAK inhibitors in patients with STAT1 GOF mutations have not been described. Therefore, we monitored the clinical and immunological changes caused by JAK inhibitor treatment in patients with STAT1 GOF mutations and used clinical immunological methods as well as plasma proteomics (proximity extension assay, Olink) and multiparametric flow analysis of immune cells by mass cytometry. The aim was to evaluate clinical and immunological changes during treatment with JAK inhibitors.

## Methods

### Clinical Data

Patient data was gathered during admission to the Department of Infectious Diseases at the Karolinska University Hospital, at Uppsala University Hospital and from medical records of Umeå University Hospital, Sahlgrenska University Hospital and Region Västernorrland, Sweden. Informed consent was obtained from all three patients.

### Radiology

Chest X-ray (CXR), computer tomography scan (CT-scan), and cerebral angiography were performed according to clinical routine at the Departments of Radiology in either Karolinska University Hospital, Umeå University Hospital, or Region Västernorrland.

### Laboratory Analyses

**Fig. 1:**
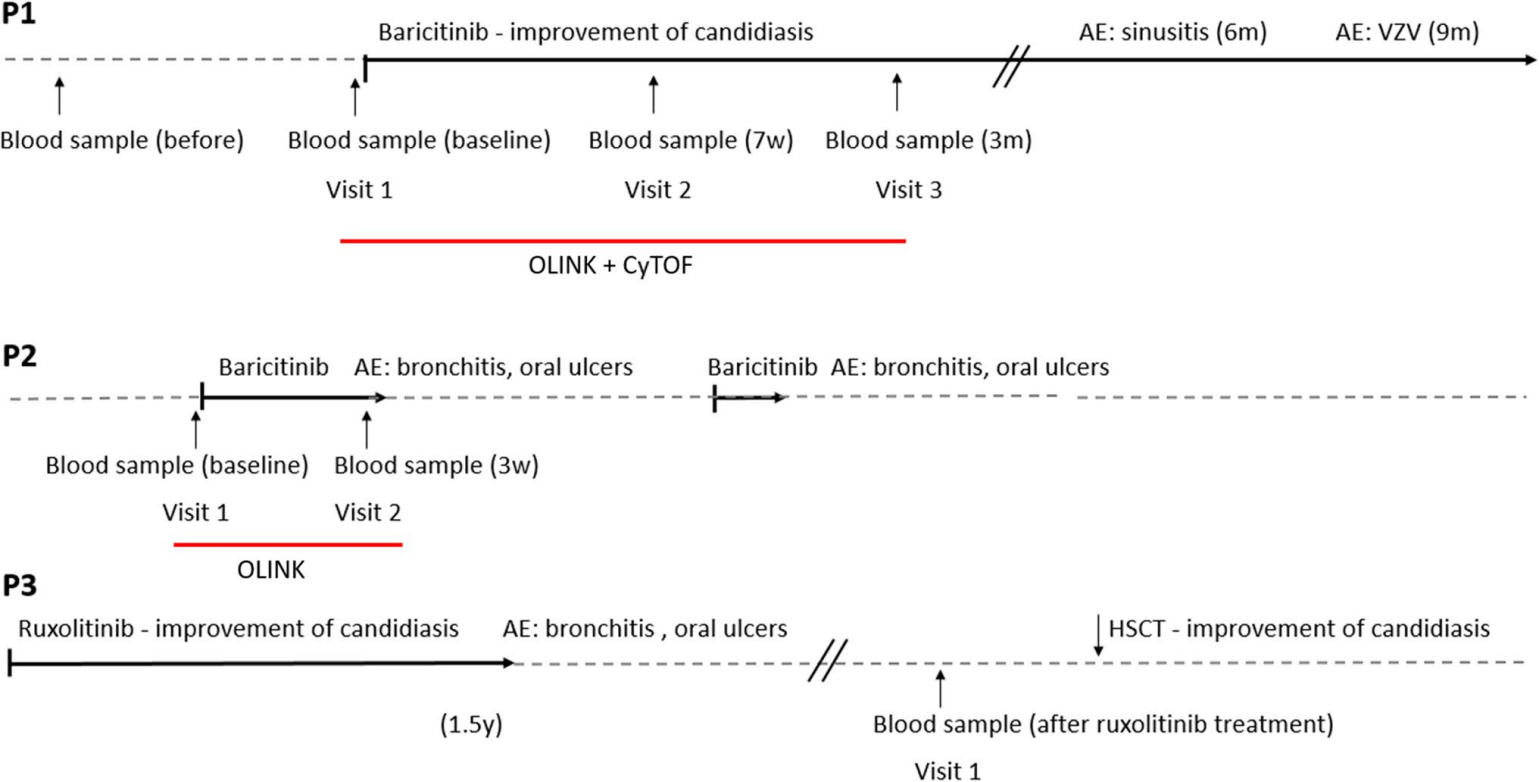
**Timetable of clinical data from three patients with CMC treated with the JAK inhibitors baricitinib and ruxolitinib** AE, adverse effect; HSCT, hematopoietic stem cell transplantation. Patient 1 (P1) was treated with baricitinib without complications and left consecutive blood samples, as indicated. Patient 2 (P2) started with baricitinib but discontinued after 3 weeks due to bronchitis and oral ulcers. Only two consecutive blood samples were collected. Patient 3 (P3) started with ruxolitinib but had to discontinue due to respiratory infections and oral ulcers. He was later subjected to HSCT, which improved his condition

Thirty milliliters of peripheral blood specimens were collected from the patients at baseline and follow-up visits for P2 and P3, during baricitinib treatment (Fig. 1).

Blood chemistry analyses were performed, as well as an immunological workup consisting of immunoglobulin levels, complement defect screening, and basic and advanced lymphocyte phenotyping. The samples were analyzed at Karolinska University Laboratory, according to clinical routines. Flow-cytometric Assay for Specific Cell-mediated Immune-response in Activated whole blood (FASCIA) was performed as previously described [43] and gating strategies are presented in Supplementary Fig. 1.

DNA analyses were performed at the Karolinska University Laboratory by whole-exome sequencing and Sanger sequencing, performed on DNA extracted from whole blood, using BigDye Terminator v.3.1 Cycle Sequencing Kit (Applied Biosystems).

### Mass Cytometry

Mass cytometry is a single-cell cytometry method based on atomic mass spectrometry allowing for multiparametric analyses. The assay was performed at Cellular Immunomonitoring Facility at SciLifeLab, Stockholm, as previously described [44–46]. The single-cell clusters were defined by a community detection algorithm *Leiden* [47]. The clusters and embeddings were generated according to PAGA (partition-based graph abstraction) pipeline [48]. In brief, a *k*-nearest neighbour’s algorithm (*k*-NN) network is first constructed where each cell is connected to its k (in our case *k* = 10) nearest neighbours according to the Euclidean distance of their markers. In this network, some cells are strongly connected compared to others and they are called a community or a cluster. After the definition of clusters, PAGA is used to define the connection of all clusters. In order to generate a single-cell embedding, we then used ForceAtlas2, a graph layout algorithm [49] with the PAGA network as the initial position.

### Proximity Extension Assay on Plasma Samples

Proximity extension assay (PEA; Olink Bioscience, Uppsala, Sweden) was performed on plasma, according to the manufacturer’s instructions [50] at Karolinska Institutet. By using three precision proteomic panels (inflammation, immune response, and Oncology II), a total of 276 biomarkers were studied simultaneously, of which 265 were unique between the panels. A complete list of all the biomarkers that were analyzed is provided in Supplementary Table 1 [51]. One µl of plasma was used for each measurement and triplicates were run for each sample. Results show the alterations in plasma proteins before and after treatment, highlighting the biomarkers that altered more than twofold. The normalized protein expression (NPX) values from the Olink assay were row-normalized for display (compared to controls) [52]. The row-normalization as well as the figures were produced using R 3.6.0, (Supplementary Figs. 2 and 3).

### Statistical Methods

Statistical analyses for patient data were not performed due to the small sample size.

## Results

### Clinical Presentation and Molecular Diagnosis

Here we present three adult patients with CMC (clinical data is presented in Table 1) referred to the Immunodeficiency Unit at the Karolinska University Hospital, Huddinge. Patient number 1 (P1) is the daughter of P2, whereas P3 is unrelated.

**Table 1:** Clinical presentation and hematological variables without JAK inhibitor treatment in three patients with chronic mucocutaneous candidiasis

	P1	P2	P3	
Age (age at genetic diagnosis)	36 (29)	60 (53)	27 (21)	
Sex	Female	Female	Male	
CMC	Yes	Yes	Yes	
Hypothyroidism	Yes	Yes	Yes	
Coeliac disease	Yes	Yes	Yes	
Atrophic gastritis	Yes	Yes	Yes	
Diabetes mellitus	No	No	Yes	
Mb Crohn	No	No	Yes	
Cerebral aneurysm Radiology	No	No	No	
CT scan thorax	Normal	Bronchiectasis	Bronchiectasis	
Laboratory data				Reference levels
Hemoglobin	130	134	134	117–153 g/L
WBC	5.9	8.1	16.4*	3.5–8.8 × 10(9)/L
Neutrophils	2.0	6.0*	14.4*	1.6–5.9 × 10(9)/L
Eosinophils	0.2	< 0.1	0.1	0–0.5 × 10(9)/L
Basophils	< 0.1	0.2*	< 0.1	0–0.1 × 10(9)/L
Lymphocytes	1.6	1.4	0.5*	1.1–3.5 × 10(9)/L
Monocytes	0.5	0.5	0.8	0.2–0.8 × 10(9)/L
Thrombocytes	286	466*	354*	145–348 × 10(9)/L
CRP	< 1	3*	12*	< 3 mg/L
S-IgG	13.9	7.9	11.5	6.7–14.5 g/L
S-IgA	4.36	1.2	1.01	0.88–4.5 g/L
S-IgM	1.06	0.33	1.17	0.27–2.1 g/L
C3	0.94	1.27	1.09	0.77–1.62 g/L
C4	0.2	0.29	0.22	0.12–0.33 g/L

*Abbreviations: P* patients, W*BC* white blood count, *ND* not done, *CRP* C-reactive protein, *IgG, IgA, IgM* serum immunoglobulin A, G and M, *C3 and C4* complement factors 3 and 4

^*^Laboratory results out of normal range. Immunoglobulin levels for P3 were measured during subcutaneous immunoglobulin replacement therapy

Whole-exome sequencing was performed in all patients. In P1 and P2, a heterozygous GOF missense alteration c.800C > T in *STAT1* (p.Ala267Val) was detected [53]. In P3, a heterozygous GOF alteration in *STAT1* (c.881 T > C, p.Ile294Thr) was found. The latter substitution has been reported to cause a combined immunodeficiency [54].

### STAT1 GOF Alterations Cause Susceptibility to Fungal, Bacterial, and Viral Infections

All three patients had recurrent fungal dermatitis and chronic mucocutaneous candidiasis. *C. albicans* was cultured repeatedly from mucus membranes and was treated with peroral antifungal therapy (fluconazole or nystatin), with the addition of intravenous voriconazole, echinocandins, or amphotericin B during flares. Voriconazole induced severe mucous membrane inflammation in P1 and P2, which prevented further use of this drug.

All three patients had increased susceptibility to bacterial airway infections of sinuses and bronchi (*Haemophilus influenzae*, *Streptococcus pneumoniae*, *Moraxella catharralis*), and P2 and P3 had developed bronchiectasis, colonized by *Pseudomonas aeruginosa.*

P1 and P2 had previously received subcutaneous immunoglobulin replacement therapy for limited periods (1–2 years), at a time. P3 had a more severe phenotype and was treated with prophylactic cephalosporin and immunoglobulin replacement therapy from young age.

A severe dermal infection (*Streptococcus pyogenes*) in P1 caused septicemia and multiple organ failure. P3 also suffered from recurrent dermal infections (*Staphylococcus aureus*) and infections of the gastrointestinal tract (*Clostridium difficile*, *Campylobacter jejuni*, and *Salmonella species*).

P2 and P3 suffered from recurrent varicella zoster infections (PCR verified), which were treated with peroral valacyclovir. All three patients tested negative for hepatitis (HBV, HCV) and HIV.

Thus, all patients exhibited increased susceptibility to infections, including fungal, bacterial, and viral species, illustrating the broad immunological impact of STAT1 GOF mutations.

### STAT1 GOF Alterations Are Associated with Severe Autoimmunity

All three patients presented with autoimmune manifestations, such as aphthous ulcers and hypothyroidism. P3 also had insulin-dependent diabetes mellitus type 1. Gastrointestinal symptoms may occur in patients with STAT1 GOF-mutations, and endoscopies performed showed intraepithelial lymphocytosis in the duodenum of P1 and P2, atrophic gastritis in P2 and P3, and a severe inflammatory bowel disease starting in childhood for P3, with deep ulcerations in the esophagus and throughout colon.

P3 was treated with immunomodulators from a young age, and later with biologics (adalimumab, ustekinumab, infliximab). However, in 2020, his clinical state deteriorated with severe gastrointestinal symptoms leading to malnutrition and he was admitted to his local hospital. A thorough clinical investigation did not reveal the underlying cause of these symptoms. An investigation to perform HSCT was initiated, as proposed in patients with a combined immunodeficiency and life-threatening disease [41, 55].

### CMC Management: JAK Inhibitor Treatment Can Be Sufficient but Transplantation May Be an Option

After a clinical assessment of P1, peroral treatment with baricitinib 2 mg/day (0.03 mg/kg) was initiated in 2019, with the addition of prophylactic fluconazole and valacyclovir. Neither prophylactic antibiotics nor immunoglobulin replacement therapy was considered necessary at that time. P1 has since then been adherent to baricitinib, except during pregnancy [40]. The treatment caused significant improvement of the mucocutaneous inflammation within a month. The aphthous ulcers subsequently healed after a period of 6 months and fluconazole and valacyclovir were discontinued. Suspected adverse events were a sinusitis with *H. influenzae* and a varicella zoster infection, which were both treated without further complications. Preventive treatment with valacyclovir was reinserted.

In P2, baricitinib treatment 2 mg/day (0.03 mg/kg) was initiated in 2020, as well as fluconazole and valacyclovir. P2 declined offered antibiotic treatment and immunoglobulin replacement therapy. There was an initial slight reduction of mucosal inflammation, but after 3 weeks, the baricitinib treatment was discontinued due to adverse effects with painful aphteous ulcers and a productive cough, fever (38 °C), aching muscles, and elevated liver enzymes. CXR showed recent bilateral peribronchial infiltration and thickened bronchial walls. The bronchitis was treated with antibiotics. The patient made another attempt with baricitinib treatment 6 months later, after receiving echinocandin treatment and prophylactic antibiotics, but again discontinued the treatment due to aphteous ulcers and increased respiratory tract symptoms. Today (May 2022), P2 still has chronic bronchitis, uses peroral preventive treatment for fungal and viral infections, and has relapses of mucocutaneous candidiasis treated with intravenous echinocandins.

P3 was previously treated (2017–2019) with ruxolitinib 15 mg/day (0.2 mg/kg/day) and was adherent for 1 and ½ years (Umeå University Hospital). The initial effect on *Candida* infections and inflamed mucus membranes was promising, but after a year, recurring lower respiratory tract infections with *S. aureus* and colonization with *P. aeruginosa* appeared. The general condition worsened over time with a chronic bronchitis, fever, and recurrence of oral candidiasis and the patient discontinued ruxolitinib treatment. The patient was offered baricitinib treatment at our clinic in 2020 but declined, because of previous experiences with ruxolitinib. In March 2021, HSCT was performed at Sahlgrenska University Hospital, Gothenburg. The conditioning therapy given was treosulfan (days − 6 to − 4), fludarbin (days − 6 to − 2), and graft-versus-host disease (GVHD)-prophylaxis with methotrexate and cyklosporin A (CyA). P3 received stem cells from a voluntary donor who was 10/10 HLA matched and blood group compatible (A +). Engraftment data showed neutrophils day + 16 and platelets day + 27. P3 was discharged at day + 36 post HSCT. One year post-transplantation, the patient’s clinical status has improved considerably, with no signs of *C. albicans*, nor GVHD, nor gastrointestinal symptoms. CyA treatment is slowly being reduced as planned. P3 is now working fulltime and states that he never felt this health since childhood.

### Clinical Flow Cytometry Data Before and During Baricitinib Treatment

Lymphocyte levels without ruxolitinib showed reduced frequencies of CD4^+^ T cells and B cells in P3 (Table 2). In addition, P2 and P3 exhibited reduced frequencies of switched memory B cells (IgD^−^CD27^+^). Low absolute counts of Th17 cells were observed in all three patients, most pronounced for P3 (Supplementary Table 2). White blood cell counts from P1 before and during treatment with baricitinib were unaltered, including total B, T, and NK cell levels (Table 3).

**Table 2:** Lymphocyte levels and subsets from patient number 1, 2, and 3 without JAK inhibitor treatment

Lymphocyte subsets	P1	P2	P3	Reference values
CD19^+^	0.15	0.35	0.02*	0.09–0.4 × 10(9)/L
IgD-CD27^+^	8	< 0.5*	1*	8–29%
CD3^+^	1.28	1.2	0.44*	0.78–2.07 × 10(9)/L
CD4^+^	0.64	0.79	0.12*	0.49–1.34 × 10(9)/L
CD8^+^	0.53	0.26	0.21	0.19–0.80 × 10(9)/L
CD4^+^CD25^+^CD127^−^	ND	4*	3*	5–11%
CD4^+^CM	25	74*	50*	14–48%
Th1/CD4^+^CM	37*	60*	48*	13–30%
Th2/CD4^+^CM	19	16	40	14–53%
Th17/CD4 + CM	13*	3*	1*	20–37%
CD4^+^EM	39	2*	23	10–47%
Th1/CD4^+^EM	40*	NA	NA	44–79%
Th2/CD4^+^EM	3*	NA	NA	4–27%
Th17/CD4^+^EM	4*	NA	NA	8–28%
NK/CD16^+^/56^+^	0.11	0.31	0.10	× 10(9)/L

Lymphocyte levels and subsets were analyzed by flow-cytometry. *P* patient, *JAK* Janus tyrosine kinase, *NA* not assessable, *ND* not done. The following cell surface markers were used to identify B-lymphocytes: CD19, T lymphocytes; CD3, T helper cells; CD4, T cytotoxic cells; CD8, NK cells; CD16/56, switched memory B lymphocytes; IgD-CD27^+^, naïve T cells; IgD^+^CD27^−^, regulatory T lymphocytes; CD4^+^CD25^+^CD127^−^, *CM* central memory, *EM* effector memory. The bloodsample for P1 is from 2015, and for P2 and P3 from 2020. *Laboratory results out of normal range

**Table 3:** White blood cell counts from patient number 1—before and during treatment with baricitinib

Blood cells	Before**	7 weeks	3 months	Ref values
WBC	5.9	4.2	5.2	3.5–8.8 × 10(9)/L
Neutrophils	2.0	1.8	3.2	1.6–5.9 × 10(9)/L
Eosinophils	0.2	< 0.1	0.1	0–0.5 × 10(9)/L
Basophils	< 0.1	< 0.1	< 0.1	0–0.1 × 10(9)/L
Lymphocytes	1.6	1.7	1.3	1.1–3.5 × 10(9)/L
Monocytes	0.5	0.5	0.5	0.2–0.8 × 10(9)/L
CD19^+^	0.15	0.18	0.15	0.09–0.4 × 10(9)/L
CD3^+^	1.28	1.29	1.03	0.78–2.07 × 10(9)/L
CD4^+^	0.64	0.68	0.55	0.49–1.34 × 10(9)/L
CD8^+^	0.53	0.51	0.42	0.19–0.80 × 10(9)/L
NK/CD16^+^/56^+^	0.11	0.09	0.07	0.07–0.42 × 10(9)/L

*Abbreviations: P* patients, *JAK* Janus tyrosine kinase, *WBC* white blood count, *ND*, not done. The following cell surface markers were used to identify B-lymphocytes: CD19, T lymphocytes; CD3, T helper cells; CD4, T cytotoxic cells; CD8, NK cells; CD16/56, switched memory B lymphocytes; IgD-CD27^+^, naïve T cells; IgD^+^CD27^−^, regulatory T lymphocytes; CD4^+^CD25^+^CD127^−^, *CM* central memory, *EM* effector memory. *Laboratory results out of normal range. **The blood sample from before baricitinib treatment was from 2015, and the two following blood samples from 2019

The functional response to *Candida* antigen was assessed by FASCIA, which quantifies CD4^+^ and CD8^+^ T cell blasts after 7 days of incubation with specific antigens. Notably, the response to *Candida* increased 7 weeks after baricitinib treatment was initiated, but normalized again after 3 months of treatment (Table 4).

**Table 4:** Specific T cell responses in patient number 1 before and during JAK inhibitor treatment (baricitinib)

Cell	Antigen stimulation	Baseline	7 weeks	3 months	Reference levels
CD4^+^	PWM	980	1853	1362	233–2189 cells/µL
CD8^+^	PWM	238	448	173	50–549 cells/µL
CD4^+^	Candida	472	2631*	547	51–1014 cells/µL
CD8^+^	Candida	81*	174*	6	0–49 cells/µL

*JAK* Janus tyrosine kinase. The following cell surface markers were used to identify T helper cells: CD4, T cytotoxic cells; CD8, *PWM* pokeweed mitogen. Whole-blood was stimulated for 7 days with specific antigens and the total number of cells/µL blood was calculated by flow-cytometry (FASCIA method). *Laboratory results out of normal range

### Immunological Changes by Mass Cytometry During Treatment with Baricitinib in Patient 1

**Fig. 2:**
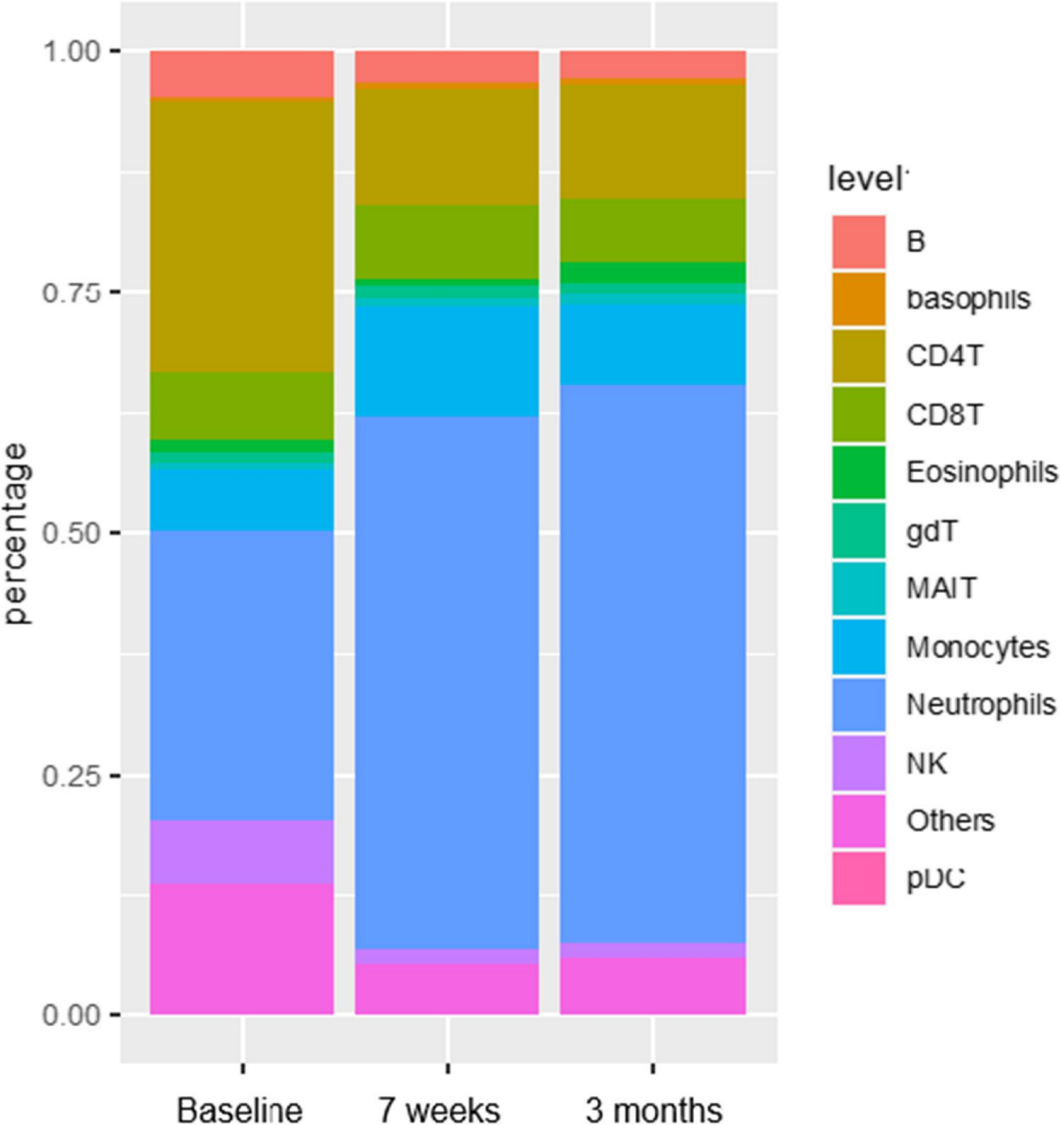
**Cell composition of patient 1 at baseline and during baricitinib treatment** The analyses were performed by mass cytometry. B, B cells; CD4T, CD4^+^ T cells; CD8, CD8^+^ T cells; gdT, gamma-delta T cells; MAIT, mucosal associated invariant T cells; NK, natural killer cells; pDC, plasmacytoid dendritical cells. The analyses were performed by mass cytometry

Blood collected from P1 before baricitinib treatment (visit 1), after 7 weeks (visit 2), and 3 months (visit 3) were assessed by mass cytometry (Fig. 2).

The percentage of naive CD4 + T cells decreased, whereas the levels of effector memory CD4 + T cells, central memory CD4 + T cells, and regulatory T cells increased. These levels were maintained at visits 2 and 3. The percentage of eosinophils decreased slightly after visit 1 and then increased at visit 3.

**Fig. 3:**
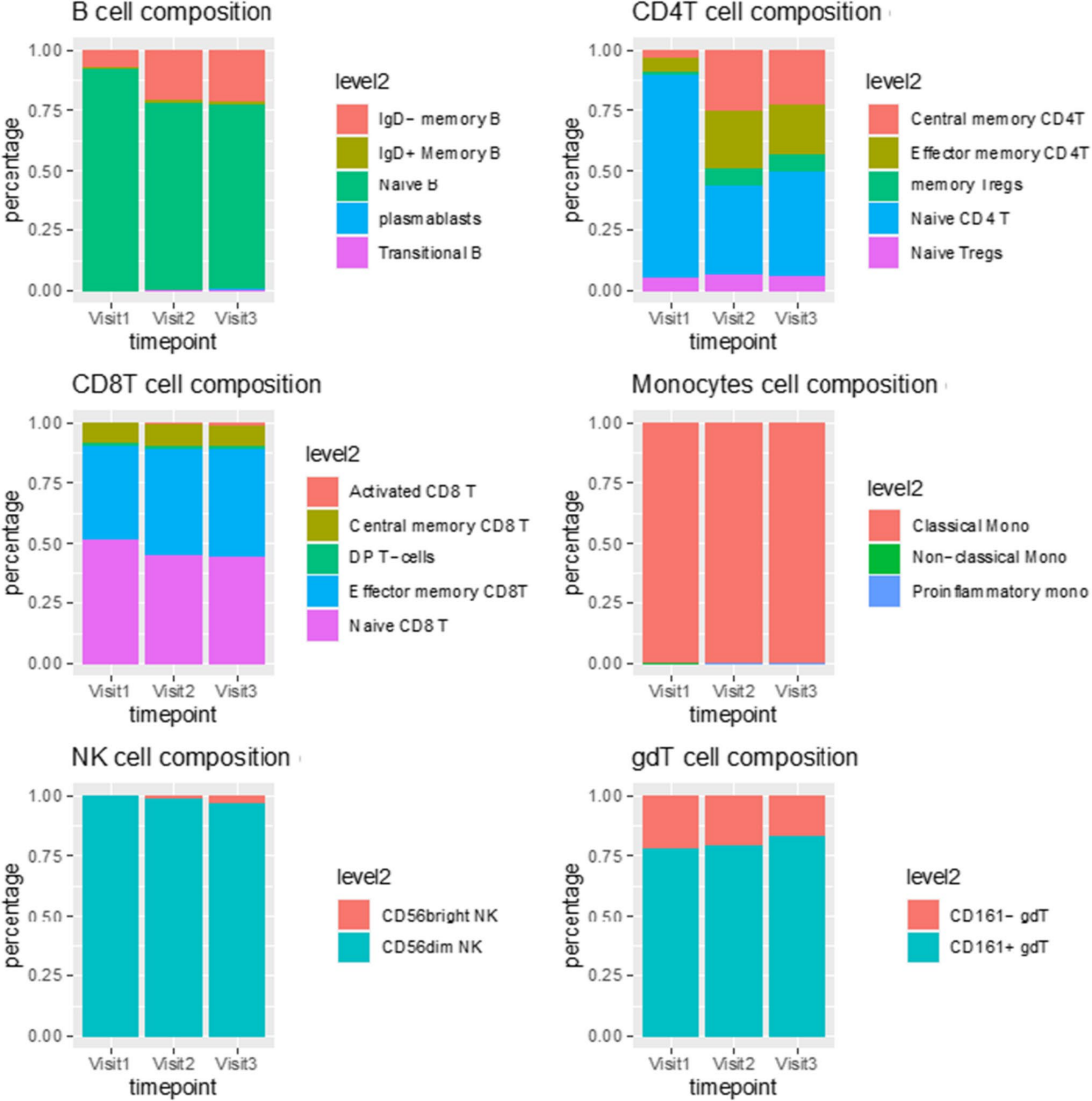
**Mononuclear subsets of patient 1 at baseline and during baricitinib treatment** Visit 1 (baseline), visit 2 (at 7 weeks), and visit 3 (at 3 months). The analyses were performed by mass cytometry. B, B cells, CD4T, CD4^+^ T cells; T regs, regulatory T cells; CD8, CD8^+^ T cells; DP T cells, CD4^+^CD8^+^ double positive T cells; gdT, gamma-delta T cells; MAIT, mucosal associated invariant T cells; NK, natural killer cells

The subtypes of different cells were measured at baseline and during treatment (Fig. 3). Late memory B cells expanded after visit 1 and naive B cells decreased, and both these cell types maintained stable at visits 2 and 3. Classical monocytes remained stable at all three visits, and NK cells expressed increased number of CD56^+^ markers at visits 2 and 3. The percentage of naive CD8^+^ T cells decreased, and a higher number of effector CD8^+^ T cells were present. The percentage of naive CD4^+^ T cells decreased, whereas the levels of effector memory CD4^+^ T cells, central memory CD4^+^ T cells, and regulatory T cells increased.

**Fig. 4:**
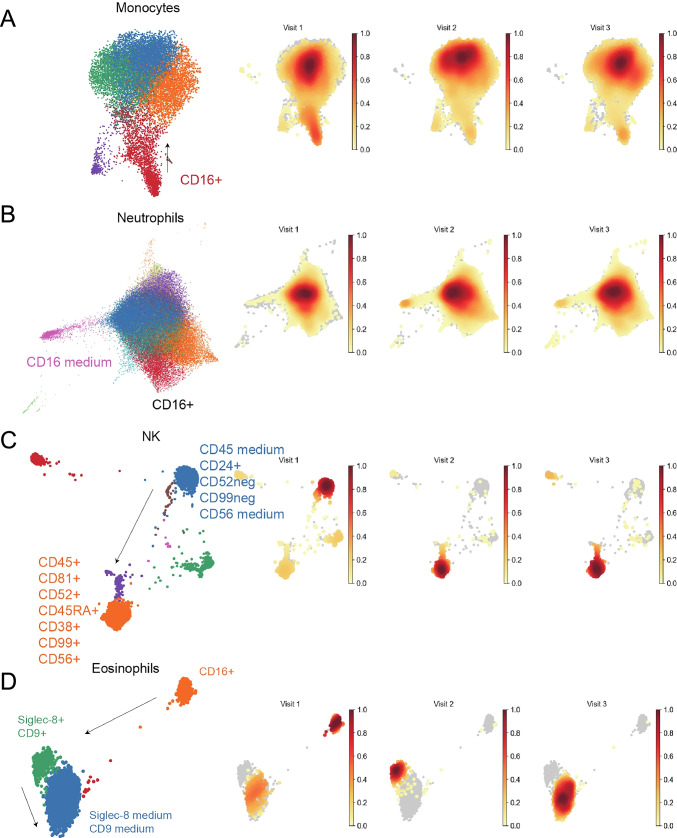
**Phenotype changes of innate immune cells in patient 1 at baseline and during baricitinib treatment** The analyses were performed by mass cytometry. Panels **A**–**D** show phenotypes of monocytes, neutrophils, natural killer cells (NK), and eosinophils and the arrows indicate the cell surface marker changes over time. The plots on the right are density plots where light-yellow color represents a relatively low cell abundance in this visit. Dark red color represents a relatively high cell abundance. The marker expression changes are assigned manually

**Fig. 5:**
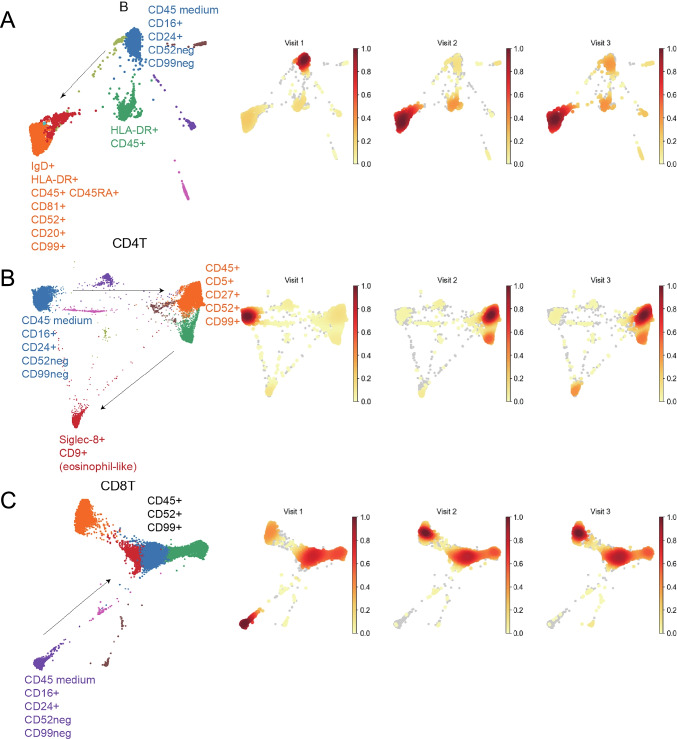
**Phenotype changes of adaptive immune cells in patient 1 at baseline and during baricitinib treatment** The analyses were performed by mass cytometry. Panels **A**–**C** show phenotypes of different subgroups of lymphocytes: CD19^+^ B cells (B), CD4^+^ T cells (CD4T), and CD8.^+^ T cells (CD8T). Arrows indicate the cell surface marker changes over time. The density plots and marker expression changes are made as described in Fig. 2

Next, cells collected at baseline, 7 weeks, and 3 months after initiating treatment in P1 were further analyzed for phenotypic changes with seven major celltypes using a panel of cellular markers, which created a high resolution map of baricitinib-induced changes. For innate cells, the most pronounced changes were observed for NK cells, where a more functional phenotype appeared after treatment, as shown by increased expression of CD45, CD52, and CD99. Monocytes and eosinophils downregulated CD16, whereas neutrophils did not exhibit major phenotypic alterations (Fig. 4A, B, C, D). Major phenotypic changes were shown in B-, CD4^+^- and CD8^+^ T cell populations, as demonstrated by increased expression of CD52, CD81, and CD99 (Fig. 5A, B, C).

A cluster of siglec-8^+^ and CD9^+^ eosinophils appeared at the 7-week visit. From visit 2 to visit 3, the levels of siglec-8^+^ and CD9^+^ cells decreased from a bright to a medium signal.

### Immunological Changes by Olink Assay During Treatment with Baricitinib in Patient 1

Finally, plasma from baseline, 7 weeks, and 3 months after treatment in P1 was analyzed for protein changes by the Olink assay enabling the analysis of 265 soluble markers (Table 5). For P1, early changes involved a twofold downregulation of CXCL10, annexin A1, granzyme B, granzyme H, and oncostatin M, whereas FGF21 was the only marker that was upregulated by more than twofold after 7 weeks. After 3 months, an upregulation of more than twofold of IL-5, VIM, 4EBP1, and PPY was observed, whereas IFN-ɣ, CCL19, and CXCL10 were downregulated (Supplementary Fig. 2). For P2, who suffered from a bacterial bronchitis during baseline and 7 weeks, all markers with at least a twofold change in protein expression, showed upregulation after 3 weeks, except for Annexin A1, ENRAGE, and S10004, which were downregulated (Supplementary Fig. 3).

**Table 5:** Selected plasma protein levels in patient number 1 that show a variation of at least twofold when comparing levels before and during treatment with baricitinib

Protein	Baseline	At 7 weeks	At 3 months
4EBP1	294	270	962
ANXA1	56	24	74
CCL19	662	340	272
CXCL10	2033	867	885
FGF21	6	16	5
GZMB	21	8	17
GZMH	50	21	70
IFN-ɣ	272	154	72
IL-5	4	5	13
OSM	25	12	27
PPY	1379	2435	2778
VIM	43	28	111

The protein levels are initially measured in triplicates (pg/µl), but are then “normalized” (median levels) in a linear graph (2^NPX^), see Olink assay *PEA* proximity extension assay. *ANXA-1* Annexin A1, *CCL19* chemokine ligand 19, *CXCL10* C-X-C motif chemokine ligand 10, *4EBP1* eukaryotic translation initiation factor 4E-binding protein 1, *FGF21* fibroblast growth factor 21, *GZMB* granzyme B, *GZMH* granzyme H, *IL5* interleukin 5, *IFN-ɣ* interferon-ɣ, *OSM* oncostatin M, *PPY* pancreatic polypeptide, *VIM* vimentin

## Discussion

### Statement of Principal Findings

Here we report three cases of chronic mucocutaneous candidiasis with infections and autoimmunity caused by STAT1 GOF alterations. The genetic diagnostics led to new treatment strategies with the JAK inhibitors baricitinib and ruxolitinib. P1 tolerated the treatment well and had a beneficial outcome (baricitinib). P2, the mother of P1, experienced adverse events in the form of aphthous ulcers and infections during treatment and had to stop (baricitinib). P3 experienced infectious complications beginning after a year of treatment, which caused cessation of the drug (ruxolitinib). Subsequently, P3 deteriorated and underwent HSCT, which improved most of the clinical symptoms. Combined, these three cases illustrate the complexity of STAT1 GOF alterations and that a personalized treatment is needed.

We also analyzed immunological variables before, during, and after treatment. As expected, the percentage of Th17 cells was low in all patients before treatment [56], and was monitored during treatment (P1), but could not be presented due to a change of the flow cytometry method at the time of the study. It should be noted that ruxolitinib has been shown to promote Th17-cell differentiation in a child with a STAT1 GOF-mutation [30]. However, another report found unaltered numbers of blood Th17 cells during JAK-inhibitor (ruxolitinib) treatment, suggesting that additional mechanisms may be involved [31].

In addition, we analyzed the cellular composition by mass cytometry and plasma markers by the Olink assay during treatment of P1. The major effect on the innate immune system was found for NK cells, which upregulated the activation markers CD45, CD52, and CD99 [57–61]. Notably, JAK inhibitor treatment has previously been shown to increase the degranulation capacity of NK cells, which could contribute to protection against viral infections and cancer in patients with STAT1 GOF [62]. Monocytes and eosinophilic granulocytes downregulated CD16, which is consistent with reduced inflammation. In addition, eosinophils upregulated the expression of siglec-8^+^ and CD9^+^, which indicate activation followed by subsequent apoptosis [63]. A case report of treatment refractory eosinophilic esophagitis associated with a STAT1 GOF alteration was recently published [64]. This report indicates an even wider phenotype than previously described, including involvement of eosinophils in the pathogenesis of STAT1 GOF mutations.

Concerning adaptive immunity, B cells upregulated several activation markers, including IgD and HLA-DR. Likewise, CD4^+^ T cells were activated at 7 weeks and later developed a siglec-8^+^ and CD9^+^ population. CD8^+^ T cells were also activated, as shown by upregulation of CD45, CD52, and CD99. The profound alterations of B- and T cell populations have only partly been described before [65], and more in-depth functional analyses are needed.

The proteomic analysis revealed that baricitinib treatment decreased the level of the chemokine CXCL10 in P1 after 3 months of treatment [66]. Notably, CXCL10 expression is dependent on *STAT1*, and patients with STAT1 GOF mutations have constitutively higher levels of this chemokine in plasma [67]. In addition, IL-5 was upregulated, and IFN-ɣ was reduced after 3 months, both of which indicate a shift from proinflammatory Th1 response to a Th2 response. Recent work by Break et al. suggested that increased IFN-ɣ signalling, through *STAT1*, disrupts the epithelial barrier. This is consistent with our finding that IFN-ɣ is decreased at 3-month post treatment which corresponds to the clinical resolution of CMC [68]. In P2, several inflammatory markers were upregulated after 3 weeks of treatment, possibly due to an infection. Interestingly, Annexin A1 levels (anti-inflammatory mediator) decreased initially in both patients, indicating an initial inflammatory phase [69].

### Strengths and Weaknesses of the Study

This study has several strengths. First, it complements the first published study on baricitinib treatment in one patient with a STAT1 GOF mutation, for whom the treatment was very successful [40]. In contrast, we show that the outcome and clinical situation may be significantly more complex due to underlying diseases in the patients, potential late adverse events, or suboptimal effects, which may necessitate HSCT. Second, we present detailed clinical and immunological data before and after treatment as well as comprehensive cellular and proteomic analyses. Combined, these data provide a possibility to study disease markers and will be hypothesis generating for future studies on this rare genetic disorder.

There are also weaknesses that need to be acknowledged. First, we only have three cases to present. They all have different disease trajectories, which complicate the reporting and make comparative analyses difficult. For example, we could only obtain a complete dataset for the mass cytometry and Olink analyses for P1, whereas P2 and P3 interrupted treatment due to side effects. As for the immunological results, many of these markers are dynamic and may be altered by other conditions such as infections, inflammation, trauma, and stress. Furthermore, we did not assess *STAT1* phosphorylation during treatment, which can be done to ensure compliance at the molecular level [40].

### Implications for Medical Treatment

Three patients with CMC are presented in this study. P1 and P2 are related and have the same GOF alteration in the *STAT1* gene. P3 has an amino acid substitution leading to a more severe clinical phenotype, as described in the literature [54]. Notably, both P2 and P3 had ongoing chronic bronchitis, while P1 did not.

All three patients were treated by JAK inhibitors (baricitinib or ruxolitinib), and all three had noticed clinical effects with a reduced general inflammation of mucous membranes. P1 was cured from CMC and has continued the treatment with baricitinib, while P2 and P3 discontinued treatment due to exacerbations of chronic bronchitis and pain from oral ulcers. Thus, it is possible that JAK inhibitors should not be administered to patients with bacterial colonization of the respiratory tract, or that an attempt to eradicate bacteria with intravenous antibiotics should be performed before JAK inhibitor treatment.

## Conclusions

Baricitinib can be a very effective treatment of mucosal inflammation in the early stages of CMC caused by STAT1 GOF mutations, before chronic pulmonary disease, and latent infections are established. Adverse effects, such as herpes virus infection, sinusitis, and bronchitis were observed, and managed for two of the patients. In one, ruxolitinib treatment was not well tolerated necessitating HSCT.

JAK inhibitors impact a broad spectrum of immune cell markers and modulate inflammatory proteins, during the healing process of CMC. In this report, we have studied 265 plasma markers using the Olink assay and a large number of cell surface markers using mass cytometry before and after treatment with JAK inhibitors. We have identified markers of both the innate and adaptive immune system that are associated with mucosal healing in this rare, genetically determined disorder, and many of these markers have never been reported previously. Because patients with STAT1 GOF variants are rare, we were only able to investigate three adult patients. In addition, our findings need to be substantiated in future studies with more patients, including patients carrying the same STAT1 GOF variants.

## Supplementary Information

Below is the link to the electronic supplementary material.

Supplementary file1 (DOCX 619 KB)

## Data Availability

The datasets generated and analyzed during the current study are available from the corresponding author on reasonable request.
