# Supplementary material for: Three Adult Cases of STAT1 Gain-of-Function with Chronic Mucocutaneous Candidiasis Treated with JAK Inhibitors
Source: J Clin Immunol. 2022 Sep 2;43(1):136–50. doi: 10.1007/s10875-022-01351-0 (PMC9840596; doi:10.1007/s10875-022-01351-0)
Supplement: Supplementary file 1 — Supplementary file1 (DOCX 619 KB) [file 10875_2022_1351_MOESM1_ESM.pdf]

Supplementary information

1. **Tables**

Supplementary Table 1. Complete list of all 265 investigated plasma biomarkers

Protein	Gene
**ABL1**	ABL1
**ADA**	ADA
**ADAM-8**	ADAM8
**ADAM-TS-15**	ADAMTS15
**ANXA1**	ANXA1
**AREG**	AREG
**ARNT**	ARNT
**ARTN**	ARTN
**AXIN1**	AXIN1
**BACH1**	BACH1
**β-NGF**	NGF
**BIRC2**	BIRC2
**BTN3A2**	BTN3A2
**CAIX**	CA9
**CASP-8**	CASP8
**CCL11**	CCL11
**CCL19**	CCL19
**CCL20**	CCL20
**CCL23**	CCL23
**CCL25**	CCL25
**CCL28**	CCL28
**CCL3**	CCL3
**CCL4**	CCL4
**CD160**	CD160
**CD207**	CD207
**CD244**	CD244
**CD27**	CD27
**CD28**	CD28
**CD40**	CD40
**CD48**	CD48
**CD5**	CD5
**CD6**	CD6
**CD70**	CD70
**CD83**	CD83
**CD8A**	CD8A
**CDCP1**	CDCP1
**CDKN1A**	CDKN1A
**CDSN**	CDSN
**CEACAM1**	CEACAM1
**CEACAM5**	CEACAM5
**CKAP4**	CKAP4
**CLEC4A**	CLEC4A
**CLEC4C**	CLEC4C
**CLEC4D**	CLEC4D

Protein	Gene
IL-18R1	IL18R1
IL-20	IL20
IL-22-Rα1	IL22RA1
IL-24	IL24
IL-10	IL10
IL-12Rβ1	IL12RB1
IL-13	IL13
IL-18	IL18
IL-2	IL2
IL-33	IL33
IL-4	IL4
IL-5	IL5
IL-6	IL6
IL-7	IL7
IL-8	CXCL8
IRAK1	IRAK1
IRAK4	IRAK4
IRF9	IRF9
ITGA11	ITGA11
ITGA6	ITGA6
ITGAV	ITGAV
ITGB5	ITGB5
ITGB6	ITGB6
ITM2A	ITM2A
JUN	JUN
KLK13	KLK13
KLRD1	KLRD1
KPNA1	KPNA1
KRT19	KRT19
LAG3	LAG3
LAMP3	LAMP3
TGF-β1	TGFB1
LIF	LIF
LIF-R	LIFR
LILRB4	LILRB4
LY75	LY75
LY9	LY9
LYN	LYN
LYPD3	LYPD3
MAD-homolog-5	SMAD5
MASP1	MASP1
MCP-1	CCL2
MCP-3	CCL7
MCP-2	CCL8

**CLEC4G**	CLEC4G
**CLEC6A**	CLEC6A
**CLEC7A**	CLEC7A
**CNTNAP2**	CNTNAP2
**CPE**	CPE
**CRNN**	CRNN
**CSF-1**	CSF1
**CST5**	CST5
**CTSV**	CTSV
**CX3CL1**	CX3CL1
**CXADR**	CXADR
**CXCL1**	CXCL1
**CXCL10**	CXCL10
**CXCL11**	CXCL11
**CXCL12**	CXCL12
**CXCL13**	CXCL13
**CXCL5**	CXCL5
**CXCL6**	CXCL6
**CXCL9**	CXCL9
**CXL17**	CXCL17
**CYR61**	CYR61
**DAPP1**	DAPP1
**DCBLD2**	DCBLD2
**DCTN1**	DCTN1
**DDX58**	DDX58
**DFFA**	DFFA
**DGKZ**	DGKZ
**DLL1**	DLL1
**DNER**	DNER
**DPP10**	DPP10
**EDAR**	EDAR
**EGF**	EGF
**EGLN1**	EGLN1
**EIF4G1**	EIF4G1
**EIF5A**	EIF5A
**EN-RAGE**	S100A12
**EPHA2**	EPHA2
**ERBB2**	ERBB2
**ERBB3**	ERBB3
**ERBB4**	ERBB4
**ESM-1**	ESM1
**FADD**	FADD
**FAM3B**	FAM3B
**FASLG**	FASLG
**FCRL3**	FCRL3
**FCRL6**	FCRL6
**FCRLB**	FCRLB
**FGF-19**	FGF19
**FGF-21**	FGF21
**FGF-23**	FGF23
**FGF-5**	FGF5
**FGF-BP1**	FGFBP1
**FGF2**	FGF2
**Flt3L**	FLT3LG

MCP-4	CCL13
MetAP-2	METAP2
MGMT	MGMT
MIA	MIA
MIC-A and MIC-B	MICA and MICB
MILR1	MILR1
MK	MDK
MMP-1	MMP1
MMP-10	MMP10
MSLN	MSLN
MUC-16	MUC16
NCR1	NCR1
NF2	NF2
NFATC3	NFATC3
NRTN	NRTN
NT-3	NTF3
NTF4	NTF4
OPG	TNFRSF11B
OSM	OSM
PADI2	PADI2
PD-L1	CD274
PIK3AP1	PIK3AP1
PLXNA4	PLXNA4
PODXL	PODXL
PPP1R9B	PPP1R9B
PPY	PPY
PRDX1	PRDX1
PRDX3	PRDX3
PRDX5	PRDX5
PRKCQ	PRKCQ
PSIP1	PSIP1
PTH1R	PTH1R
PVRL4	NECTIN4
RET	RET
RSPO3	RSPO3
S100A11	S100A11
S100A4	S100A4
SCAMP3	SCAMP3
SCF	KITLG
SEZ6L	SEZ6L
SH2B3	SH2B3
SH2D1A	SH2D1A
SIRT2	SIRT2
SIT1	SIT1
SLAMF1	SLAMF1
SPARC	SPARC
SPRY2	SPRY2
SRPK2	SRPK2
ST1A1	SULT1A1
STAMBP	STAMBP
STC1	STC1
SYND1	SDC1
TANK	TANK
TCL1A	TCL1A

**FR-α**	FOLR1
**FR-γ**	FOLR3
**FURIN**	FURIN
**FXYD5**	FXYD5
**Gal-1**	LGALS1
**GALNT3**	GALNT3
**GDNF**	GDNF
**GLB1**	GLB1
**GPC1**	GPC1
**GPNMB**	GPNMB
**GZMB**	GZMB
**GZMH**	GZMH
**HCLS1**	HCLS1
**HEXIM1**	HEXIM1
**HGF**	HGF
**hK11**	KLK11
**hK14**	KLK14
**hK8**	KLK8
**HNMT**	HNMT
**HSD11B1**	HSD11B1
**ICA1**	ICA1
**ICOSLG**	ICOSLG
**IFN-γ**	IFNG
**IFN-γR1**	IFNGR1
**IFNLR1**	IFNLR1
**IGF1R**	IGF1R
**IL-17α**	IL17A
**IL-17C**	IL17C
**IL-20Rα**	IL20RA
**IL-2Rβ**	IL2RB
**IL-1-α**	IL1A
**IL-10Rα**	IL10RA
**IL-10Rβ**	IL10RB
**IL-12β**	IL12B
**IL-15Rα**	IL15RA

TFPI-2	TFPI2
TGF-α	TGFA
TGFR-2	TGFBR2
TLR3	TLR3
TNF	TNF
TNFB	LTA
TNFRSF19	TNFRSF19
TNFRSF4	TNFRSF4
TNFRSF6B	TNFRSF6B
TNFRSF9	TNFRSF9
TNFSF13	TNFSF13
TNFSF14	TNFSF14
TPSAB1	TPSAB1
TRAF2	TRAF2
TRAIL	TNFSF10
TRANCE	TNFSF11
TREM1	TREM1
TRIM21	TRIM21
TRIM5	TRIM5
TSLP	TSLP
TWEAK	TNFSF12
TXLNA	TXLNA
uPA	PLAU
VEGFA	VEGFA
VEGFR-2	KDR
VEGFR-3	FLT4
VIM	VIM
WFDC2	WFDC2
WIF-1	WIF1
WISP-1	WISP1
4E-BP1	EIF4EBP1
5-NT	NT5E
XPNPEP2	XPNPEP2
ZBTB16	ZBTB16

Supplementary Table 2. Lymphocyte levels and subsets from patient nr 1, 2 and 3 without JAK inhibitor treatment

**Lymphocyte subsets**	**P1**	**P2**	**P3**	**Reference values**
$CD19^+$	0.15	0.35	0.02*	0.09-0.4 x10(9)/L
$IgD\text{-}CD27^+$	0.012	<0.01	<0.001	0.0072-0.11 x10(9)/L
$CD3^+$	1.28	1.2	0.44*	0.78-2.07 x10(9)/L
$CD4^+$	0.64	0.79	0.12*	0.49-1.34 x10(9)/L
$CD8^+$	0.53	0.26	0.21	0.19-0.80 x10(9)/L
$CD4^+CD25^+CD127^-$	ND	0.0316	0.0036*	0.02-0.15 x10(9)/L
$CD4^+CM$	0.16	0.58	0.06*	0.07-0.64 x10(9)/L
$Th1/CD4^+CM$	0.06	0.35	0.03	0.01-0.19 x10(9)/L
$Th2/CD4^+CM$	0.03	0.09	0.02	0.01-0.34 x10(9)/L
$Th17/CD4^+CM$	0.02	0.02	0.001*	0.014-0.24 x10(9)/L
$CD4^+EM$	0.25	0.02*	0.03*	0.05-0.63 x10(9)/L
$Th1/CD4^+EM$	0.1*	NA	NA	0.22-1.06 x10(9)/L
$Th2/CD4^+EM$	0.01*	NA	NA	0.02-0.36 x10(9)/L
$Th17/CD4^+EM$	0.01*	NA	NA	0.04-0.38 x10(9)/L
CD16/56	0.11	0.31	0.1	0.07-0.42 x10(9)/L

Data is calculated from Table 2 in actual numbers. Lymphocyte levels and subsets were analyzed by flow-cytometry. Patient (P), Janus tyrosine kinase (JAK), not assessable (NA), not done (ND). The following cell surface markers were used to identify B-lymphocytes: CD19;T-lymphocytes: CD3; T-helper cells; CD4; T-cytotoxic cells: CD8; NK-cells: CD16/56; switched memory B-lymphocytes: $IgD\text{-}CD27^+$; naïve T-cells: IgD^+CD27^- ; regulatory T-lymphocytes: $CD4^+CD25^+CD127^-$; CM (central memory); EM (effector memory). The bloodsample for P1 in this table is from 2015, and for P2 and P3 from 2020. *Laboratory results out of normal range.

2. Figures

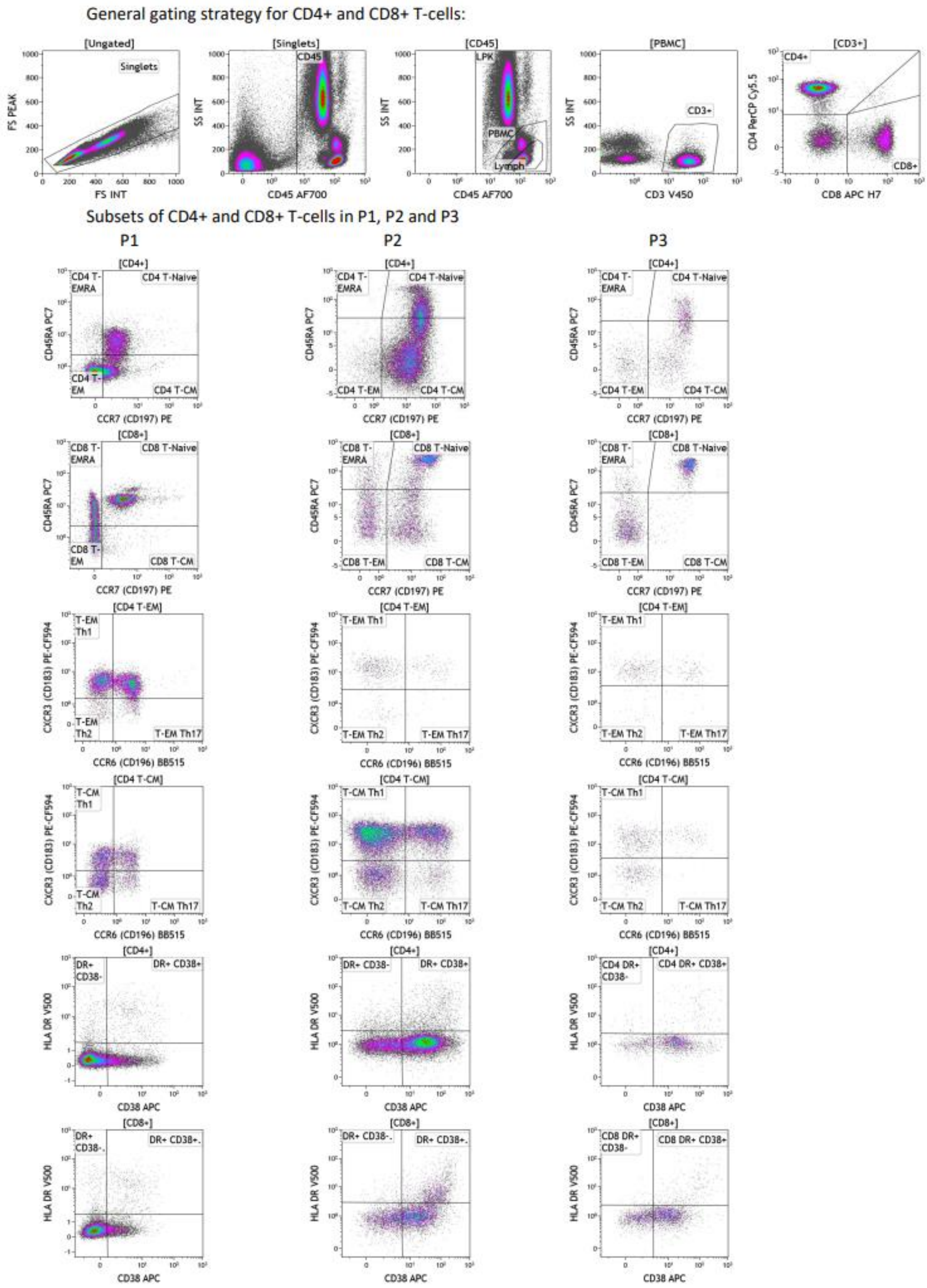

Supplementary Fig. 1 Gating strategy for $CD4^+$ and $CD8^+$ cells by flow-cytometry

In the first row the general gating strategy for $CD4^+$ and $CD8^+$ T-cells from whole-blood is presented. In the following rows central and effector memory T-cell subsets and B-cell subsets are gated in patient 1, 2 and 3 by specific cell markers. Data of <1000 events per square are considered too low to report.

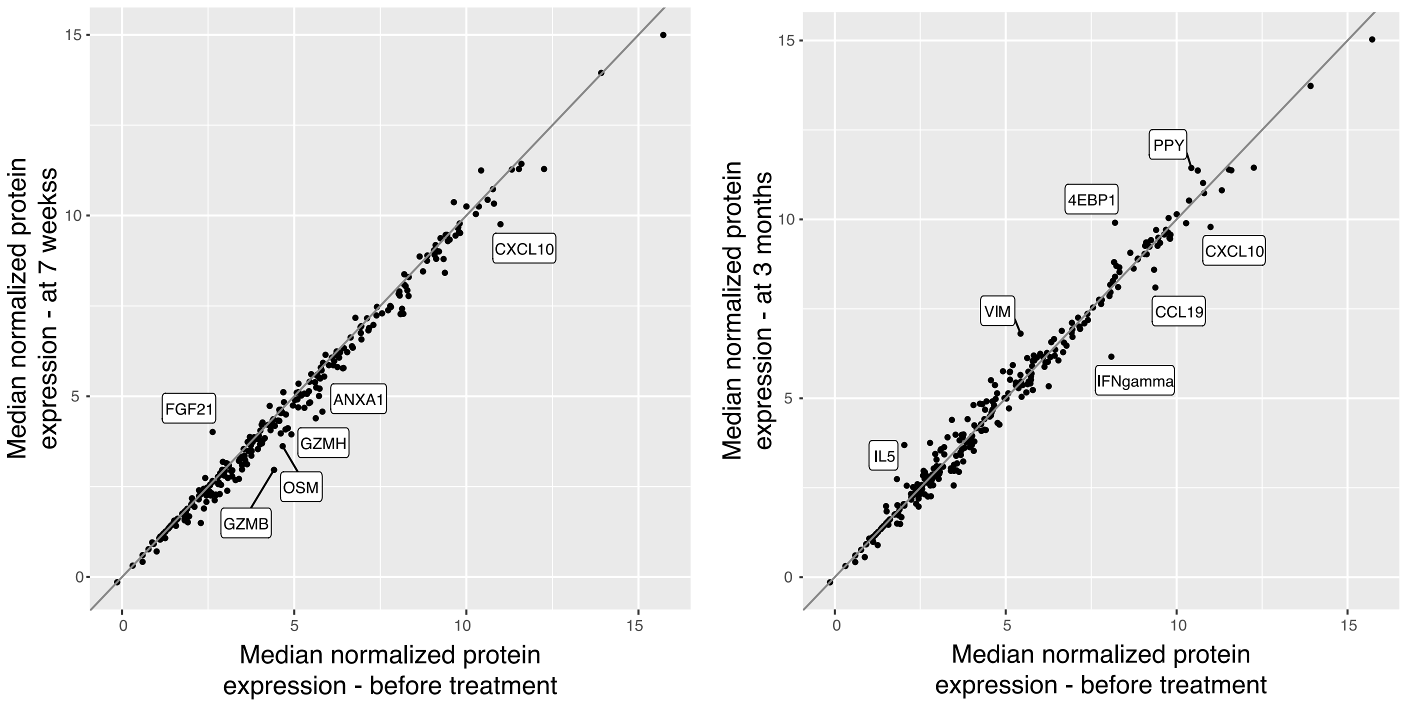

Supplementary Fig. 2 Changes in plasma proteins before and after treatment with baricitinib in patient 1 before and after 7 weeks and 3 months of treatment with baricitinib

Values are displayed in normalized protein expression (NPX) a unit that is in Log2 scale.
Analysis performed by Olink, Proximity Extension Assay=PEA. Annexin A1=ANXA-1, Chemokine ligand 19=CCL19, C-X-C motif chemokine ligand 10 = CXCL10, Eukaryotic translation initiation factor 4E- binding protein 1=4EBP1, Fibroblast growth factor 21=FGF21, Granzyme B= GZMB, Granzyme H=GZMH, Interleukin 5=IL5, Interferon-ɣ=IFNgamma, Oncostatin M=OSM, pancreatic polypeptide=PPY, Vimentin = VIM.
Scatterplots where each dot represents the median plasma biomarker levels before and after treatment, time-points compared are indicated on the axis. The diagonal line represents a fold change of zero. Plasma biomarkers with an increase or decrease of at least two-fold are labelled. Normalized protein expression is a unit in Log2 scale. The values for the labelled median plasma protein levels can be found in Table 5.

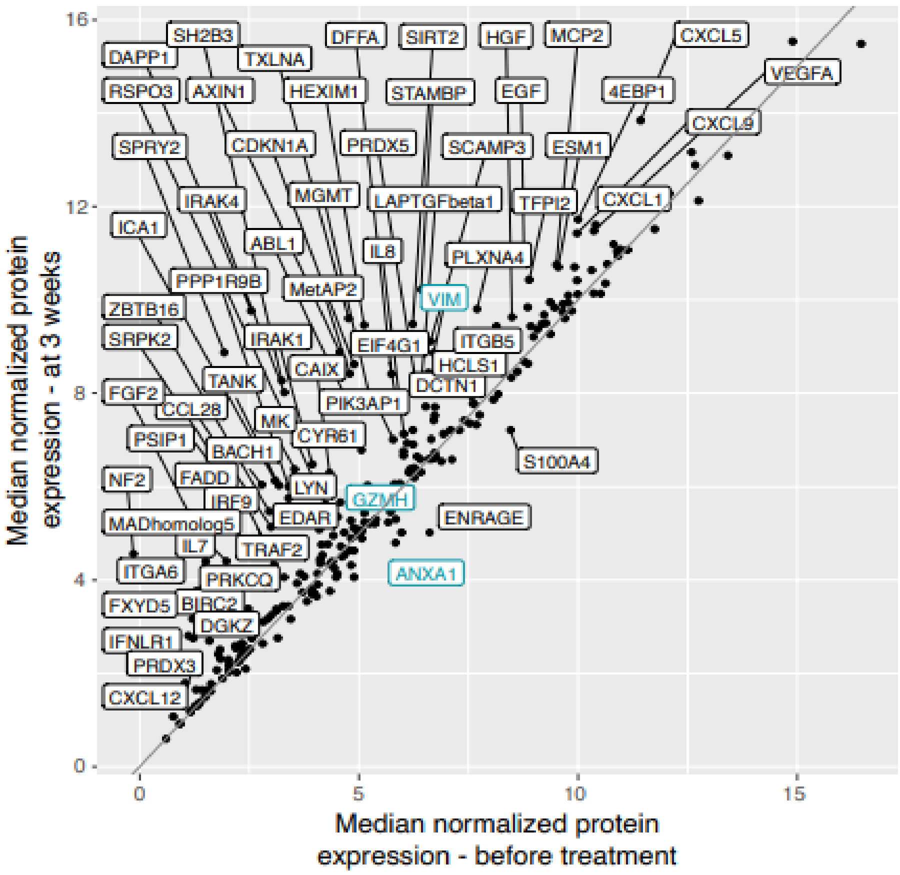

Supplementary Fig. 3 Changes in plasma proteins before and after treatment with baricitinib in patient 2 at baseline and after 3 weeks of treatment with baricitinib

Analysis performed by Olink, Proximity Extension Assay=PEA. Values are displayed in normalized protein expression (NPX) a unit that is in Log2 scale. Annexin A1=ANXA-1, Granzyme H=GZMH, Vimentin = VIM. Scatterplots where each dot represents the median plasma biomarker levels before and after treatment, time-points compared are indicated on the axis. The diagonal line represents a fold change of zero. Plasma biomarkers with an increase or decrease of at least two-fold are labelled. Normalized protein expression is a unit in Log2 scale. Proteins found in both Fig. 2 and Fig. 3 are marked in blue. The values for the labelled median plasma protein levels can be found in Table 5.
